# DNA demethylase Tet2 suppresses cisplatin-induced acute kidney injury

**DOI:** 10.1038/s41420-021-00528-7

**Published:** 2021-06-17

**Authors:** Yinwu Bao, Mengqiu Bai, Huanhuan Zhu, Yuan Yuan, Ying Wang, Yunjing Zhang, Junni Wang, Xishao Xie, Xi Yao, Jianhua Mao, Xianghui Fu, Jianghua Chen, Yi Yang, Weiqiang Lin

**Affiliations:** 1 The Kidney Disease Center, the First Affiliated Hospital, Zhejiang University School of Medicine; Institute of Nephrology, Zhejiang University; Key Laboratory of Kidney Disease Prevention and Control Technology, Hangzhou, Zhejiang Province 310003 China; 2 Department of Nephrology, The Fourth Affiliated Hospital, Zhejiang University School of Medicine, Jinhua, 322000 China; 3 Institute of Translational Medicine, Zhejiang University School of Medicine, Hangzhou, 310029 China; 4 Department of Nephrology, Children’s Hospital, Zhejiang University School of Medicine, Hangzhou, 310003 China; 5 Division of Endocrinology and Metabolism, State Key Laboratory of Biotherapy and Cancer Center, West China Hospital, Sichuan University and Collaborative Innovation Center of Biotherapy, Chengdu, 610041 China

**Keywords:** Epigenetics, Acute kidney injury, Kidney diseases

## Abstract

Demethylase Tet2 plays a vital role in the immune response. Acute kidney injury (AKI) initiation and maintenance phases are marked by inflammatory responses and leukocyte recruitment in endothelial and tubular cell injury processes. However, the role of *Tet2* in AKI is poorly defined. Our study determined the degree of renal tissue damage associated with *Tet2* gene expression levels in a cisplatin-induced AKI mice model. *Tet2*-knockout (KO) mice with cisplatin treatment experienced severe tubular necrosis and dilatation, inflammation, and AKI markers’ expression levels than the wild-type mice. In addition, the administration of *Tet2* plasmid protected *Tet2*-KO mice from cisplatin-induced nephrotoxicity, but not *Tet2*-catalytic-dead mutant. *Tet2* KO was associated with a change in metabolic pathways like retinol, arachidonic acid, linolenic acid metabolism, and PPAR signaling pathway in the cisplatin-induced mice model. Tet2 expression is also downregulated in other AKI mice models and clinical samples. Thus, our results indicate that Tet2 has a renal protective effect during AKI by regulating metabolic and inflammatory responses through the PPAR signaling pathway.

## Introduction

Acute kidney injury (AKI) is a global public health concern impacting ∼13.3 million patients per year [1]. Apparently, direct nephrotoxin is believed to be the reason for AKI development in about 20% of patients [2]. Cisplatin is an effective chemotherapeutic drug used for various solid tumors; however, AKI associated with cisplatin treatment limits its clinical usage [3]. High concentration of cisplatin accumulation has toxic effects in the proximal tubule segment [4]. Although multiple mechanisms such as ferroptosis oxidative stress injury, dysfunction of autophagy, necrosis, and apoptosis contribute to the pathogenesis of cisplatin-induced AKI, more and more evidence suggests inflammation to play a crucial role [5–9]. Cisplatin-induced AKI demonstrated an increased concentration of various pro-inflammatory cytokines such as IL-1β, IL-6, IL-18, tumor necrosis factor (TNF)-α, monocyte chemotactic protein-1 (MCP-1), and transforming growth factor-β1 (TGF-β1) [3, 10, 11].

Ten eleven translocation (Tet) methylcytosine dioxygenase family members are known to catalyze 5-methylcytosine (5mc) to 5-hydroxymethylcytosine (5hmc). Besides, Tet proteins play a role in regulating immunity and inflammation through the DNA methylation-independent way. It has also been reported that in dendritic cells and macrophages, Tet2 can suppress inflammation by recruiting histone deacetylases 2 (Hdac2) to repress IL-6 transcription specifically and downregulate other inflammatory mediators [12]. *Tet2* KO will increase the IL-1β/NLRP3 inflammasome production resulting in accelerated development of atherosclerosis and heart failure [13, 14]. *Tet2* is essential for various pathological processes, including leukemia, atherosclerotic cardiovascular diseases, and inflammation [12, 14, 15]. However, the role of Tet2 in AKI remains largely unknown. Our studies demonstrated the protective role of *Tet2* in cisplatin-induced AKI by modulating metabolic and inflammatory responses. As known, cisplatin exacerbated AKI in *Tet2*-KO mice, but administration of *Tet2* plasmid protected *Tet2*-KO mice from cisplatin-induced nephrotoxicity. In addition, renal RNA-seq results showed that *Tet2* deletion correlated with downregulation of the PPAR pathway genes’ expression in metabolism regulation and increased the expression of inflammatory cytokines.

## Results

### *Tet2* is highly expressed in mice kidney, kidney cell line and decreased in cisplatin-induced AKI

**Fig. 1:**
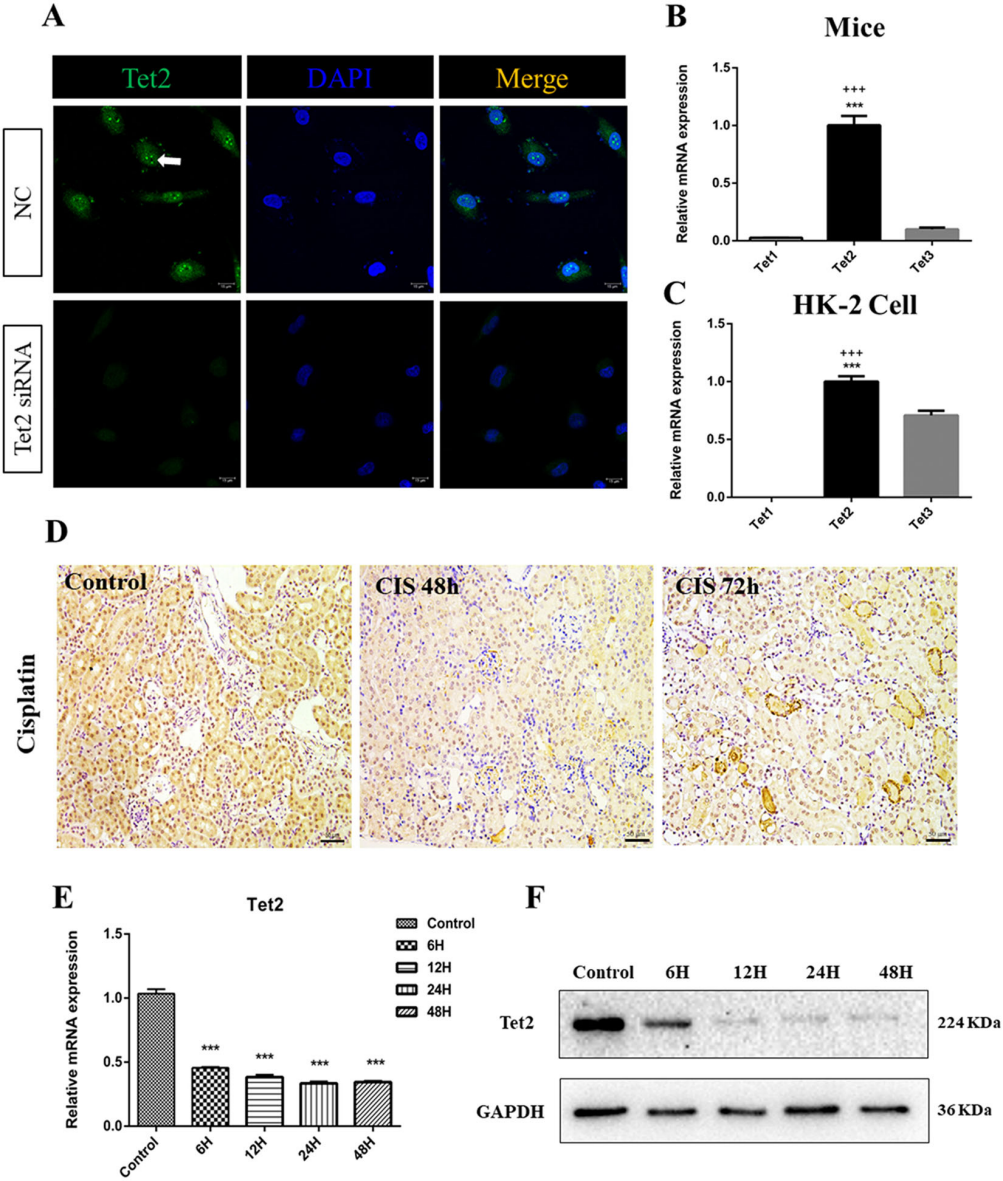
Highly expressed *Tet2* in normal kidney and renal epithelial tubular cells. **A** Immunofluorescence in HK-2 cells showing the location of Tet2 (green, orange arrows) predominantly in the nucleus (DAPI, blue), while the signal of Tet2 was attenuated by RNAi. Scale bar, 15 μm. **B**, **C** Both in vivo and in vitro qPCR analyses showing high expression of *Tet2*. ***P < 0.001 versus *Tet1* gene; ^+++^*P* < 0.001 versus *Tet3* gene (*n* = 3). **D** Male C57BL*/*6 mice intraperitoneally injected with 0.9% saline or 22 mg/kg cisplatin (*n* = 6). Immunohistochemistry revealed a reduced *Tet2* expression in the AKI model (original magnification, ×200). **E**, **F** HK-2 cells were treated with 10 µM cisplatin to mimic acute injury. **E** qPCR analyses showing a significant downregulation of *Tet2* mRNA in the cisplatin-treated HK-2 cells compared to control cells. **F** Western blot analyses demonstrate a decreased Tet2 protein in the HK-2 cells after cisplatin treatment compared to the control cells.

We first explored the in vitro and in vivo expression of the *Tet2* gene under healthy and disease conditions. We performed immunofluorescence staining of *Tet2* in HK-2 cells to examine the site and *Tet2* expression. Significant Tet2 protein enrichment was observed in the nucleus, and siRNA knockdown significantly decreased the immunofluorescence intensity (Fig. 1A). Real-time quantitative PCR (qPCR) results showed a high expression of *Tet2* mRNA in WT-mice kidney compared to *Tet1* and *Tet3* genes (Fig. 1B). Normal HK-2 cells showed similar results (Fig. 1C). To investigate the *Tet2* expression in cisplatin-induced AKI, we established an AKI mice model by intraperitoneal injection of 22 mg/kg cisplatin. The mice-kidney immunohistochemistry revealed a marked reduction in Tet2 protein after 48 and 72 h of cisplatin administration (Fig. 1D). Similar results were observed in the in vitro experiments. HK-2 cells treated with 10 µM cisplatin (6, 12, 24, and 48 h) showed ~50% *Tet2* mRNA level reduction after 6 h of cisplatin treatment. Western blot analyses also showed a dramatic reduction of cellular Tet2 protein in cisplatin-treated cells compared to the controls (Fig. 1E, F). These observations indicated that *Tet2* might play a predominant role in cisplatin-induced AKI than the other two family members (*Tet1* and *Tet3*).

### Characterization of *Tet2*-knockout mice

**Fig. 2:**
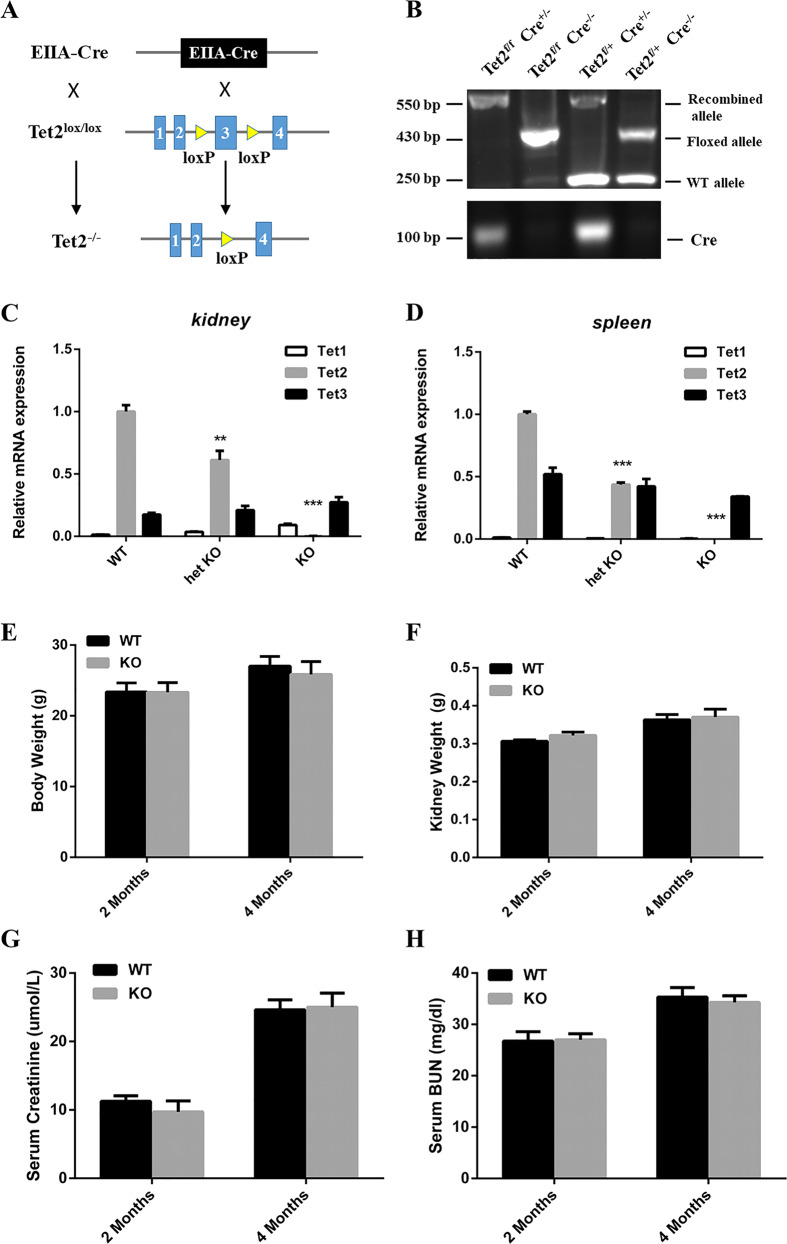
Characterization of *Tet2*-KO mice. **A** The schematic diagram showing the crossbreeding strategy of *Tet2*-floxed mice (*Tet2*^*f/f*^) with EIIa-Cre transgenic mice. **B** Mice genotyping by PCR analyses of genomic DNA. Lane 1: *Tet2*^*f/f*^, Cre^+/−^, designated as KO, Lane 2: *Tet2*^*f/f*^, Cre^−/−^, designated as WT. **C** Real-time PCR analyses demonstrated a substantial reduction of the spleen and renal *Tet2* mRNA in KO mice compared to WT mice. ***P < 0.001 versus WT mice. **E**–**H** *Tet2* gene deletion had no impact on phenotype. There was no significant difference in the body weight (**E**), kidney weight (**F**), Scr (**G**), BUN (**H**) between KO and WT mice at 2 and 4 months (*n* = 6).

To further delineate the function of Tet2 in cisplatin-induced AKI, we generated *Tet2*-KO mice by using the Cre-loxP system. Homozygous *Tet2*-KO mice were created by mating with transgenic mice expressing Cre recombinase (EIIA-Cre)—their Cre was expressed widely in the early embryo. The exon3 between the two LoxP sites in the target gene was deleted after mating with LoxP mice (Fig. 2A). Figure 2B shows the various genotypes of individual mice genomic DNA through PCR analyses. Mice with *Tet2* ablation were designated as KO (*Tet2*^*f/f*^ Cre^+/−^) (Fig. 2B, lane 1), whereas *Tet2*-floxed mice were designated WT (*Tet2*^*f/f*^ Cre^−/−^) (Fig. 2B, lane 2). The qPCR experiment results showed that the *Tet2* mRNA level reduced by 90% or more in the kidney and spleen isolated from KO mice, whereas it reduced by 40–50% in heterozygous-KO (het KO) mice compared to control-WT littermate mice. Furthermore, we also found that *Tet1* and *Tet3* mRNA levels were not affected by *Tet2* ablation, and there were hardly any alterations in *Tet1* and *Tet3* mRNA expression (Fig. 2C, D). Moreover, there was no significant difference in the mice body weight, kidney weight, serum creatinine (Scr), and blood urea nitrogen (BUN) between KO and their control littermates at 2 and 4 months after birth (Fig. 2E–H). Therefore, we excluded the phenotypic variance caused by *Tet2* gene deletion.

### *Tet2* deletion exacerbates cisplatin-induced AKI

**Fig. 3:**
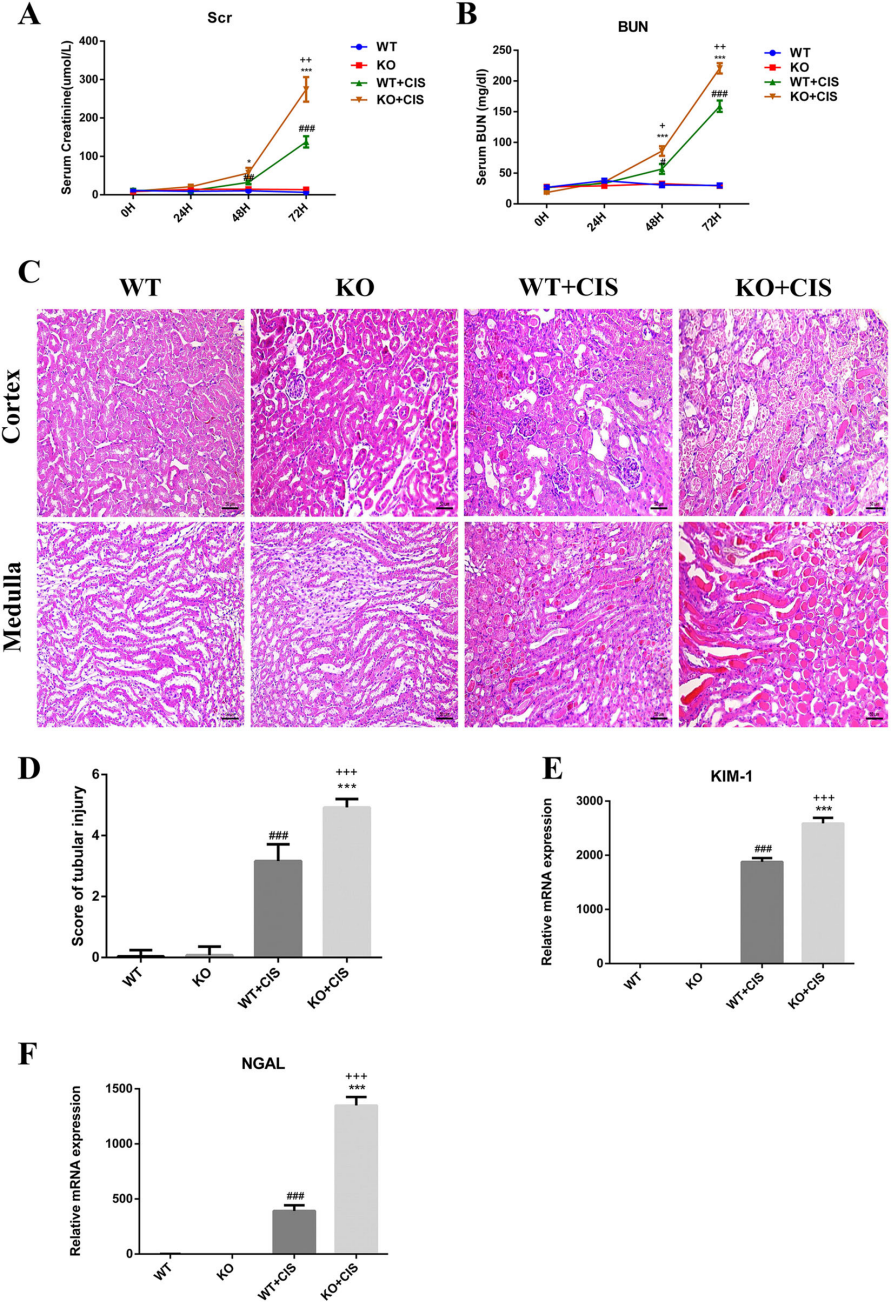
*Tet2* KO accelerates AKI progression after cisplatin injection. **A**, **B** Cispaltin treatment (22 mg/kg) led to a marked elevation of both Scr and BUN in KO + CIS mice compared to the KO and WT + CIS mice (*n* = 6). **C** Representative images of HE-stained kidney sections (200×). **D** Histologic damage in HE-stained kidney sections (*n* = 6) was scored by the criterion used in previous research. Ten randomly selected fields (original magnification, ×200) per kidney were required for counting the percentage of tubules that displayed cast formation, tubular necrosis, and dilation as follows: 0 = normal; 1 ≤ 10%; 2 = 10–25%; 3 = 26–50%; 4 = 51–75%; 5 ≥ 75%. **E**, **F** qRT-PCR analyses revealed a remarkable increase in KIM-1 and NGAL expression in KO + CIS mice, compared to KO and WT + CIS mice. *P < 0.05, **P < 0.01, and ***P < 0.001 represent statistical significance between WT + CIS mice or KO + CIS mice versus KO mice; ^#^*P* < 0.05, ^##^*P* < 0.01, and ^###^*P* < 0.001 represent statistical significance between WT + CIS mice or KO + CIS mice versus WT mice; ^+^*P* < 0.05, ^++^*P* < 0.01, and ^+++^*P* < 0.001 represent statistical significance between KO + CIS mice versus WT + CIS mice.

A cisplatin-induced AKI mice model was established by administering 22 mg/kg cisplatin to both WT and *Tet2*-KO mice to investigate the role of *Tet2*. It was observed that the Scr and BUN levels (indicative of renal function) were markedly elevated in the KO and WT mice at 48 and 72 h after cisplatin injection (Fig. 3A, B). Furthermore, it was noticed that the Scr and BUN levels increased dramatically in the KO mice than the WT mice. Histological analysis with hematoxylin–eosin (HE) staining showed an intensified cast formation, tubular necrosis, and dilation in both mice after cisplatin treatment; pathological phenotype was more severe in KO mice than WT mice (Fig. 3C, D). There was a significant increase in the expression level of kidney injury molecule-1 (KIM-1) and neutrophil gelatinase-associated lipocalin (NGAL) in the kidneys of cisplatin-injected mice; the increase was higher in the KO mice than the WT mice (Fig. 3E, F). Thus, our data confirmed that *Tet2* ablation accelerated renal function loss and aggravated kidney injury after cisplatin administration.

### In vivo expression of *Tet2* attenuates cisplatin-induced AKI

**Fig. 4:**
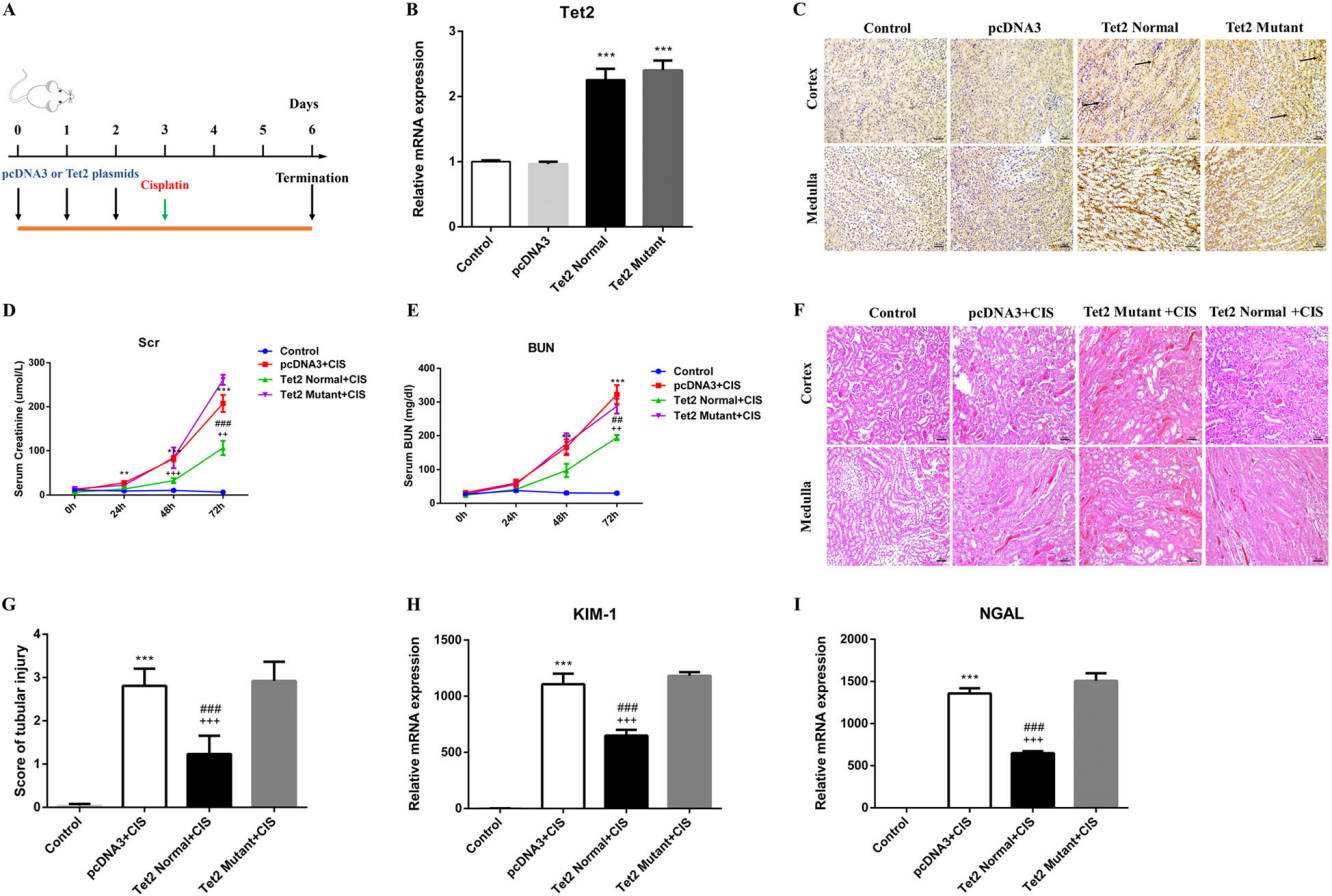
In vivo expression of *Tet2* attenuates cisplatin-induced AKI. **A** The diagram shows the experimental design. Black arrows indicate the time point of when pcDNA3 or *Tet2* plasmids were injected. The green arrowhead represents the time point of cisplatin administration (*n* = 3). **B**, **C** qRT-PCR analyses and immunohistochemistry of mouse kidney reveal an increased *Tet2* expression after plasmids injection. **D**, **E** Overexpression of *Tet2* Normal plasmid can significantly reduce the marked elevation of Scr and BUN caused by cisplatin injection. **F**, **G** Representative images of HE-stained kidney sections (*n* = 6) (200×) and histologic damage in the section were scored by the same evaluation method as shown in Fig. 4. **H**, **I** Graphic presentation shows the levels of KIM-1 and NGAL in different groups. ***P < 0.001 versus Control; ^###^*P* < 0.001 versus pcDNA3 group; ^+++^*P* < 0.001 versus *Tet2* Mutant group.

We performed in vivo introduction of *Tet2* in the kidneys during the AKI progression to further confirm the role of *Tet2* in cisplatin-induced AKI. A small amount (2 mg/kg) of mouse *Tet2* catalytic domain expression vector (*Tet2* Normal), *Tet2* catalytic domain vector with non-catalytic function (*Tet2* Mutant), or empty vector (pcDNA3) were administered intravenously through hydrodynamic gene transfer technique for 3 days before cisplatin treatment (Fig. 4A) [16, 17]. Quantitative analyses demonstrated *Tet2* induction in kidneys after continuous injection of *Tet2*-expressing plasmids (Fig. 4B). Immunohistochemistry analysis also showed similar results (Fig. 4C).

We further investigated if overexpression of *Tet2* could positively impact cisplatin-induced AKI. The Scr and BUN elevation was significantly inhibited in *Tet2*-overexpressed mice after cisplatin administration (Fig. 4D, E). The HE-stained kidney sections revealed that *Tet2*-overexpression dramatically reduced tubular injury and necrosis in cisplatin-administered treatment, while the *Tet2* Mutant or control plasmids could not (Fig. 4F, G). In addition, the qRT-PCR assessment confirmed KIM-1 and NGAL downregulation in the *Tet2* Normal plasmid injection group compared to the injection groups of pcDNA3 or *Tet2* Mutant plasmid (Fig. 4H, I).

### Deletion of *Tet2* contributes to aggravated metabolic pathway disorders and inflammation induced by cisplatin

**Fig. 5:**
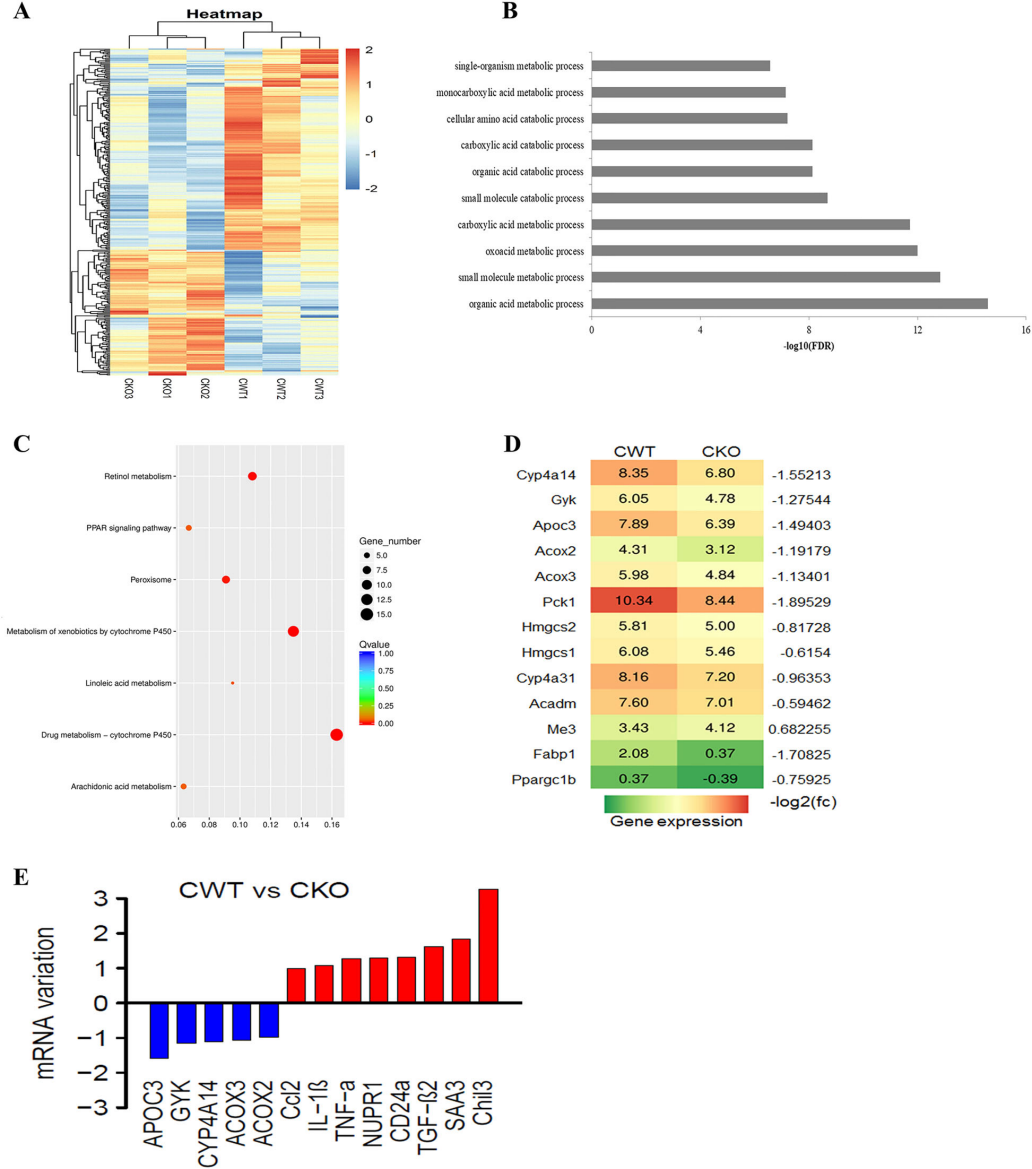
Gene expression variation in WT/*Tet2-*KO mice injected with cisplatin. **A** Different expression genes heatmap. Cisplatin treatment group: CWT vs. CKO (*n* = 3). **B**, **C** Enrichment pathways in the cisplatin treatment group. **D**, **E** mRNA variations of indicated genes in RNA-seq analysis of mice stimulated with cisplatin. RPKM of each of the genes in the CKO group were compared with the CWT group, and calculated to log2 ratio.

**Fig. 6:**
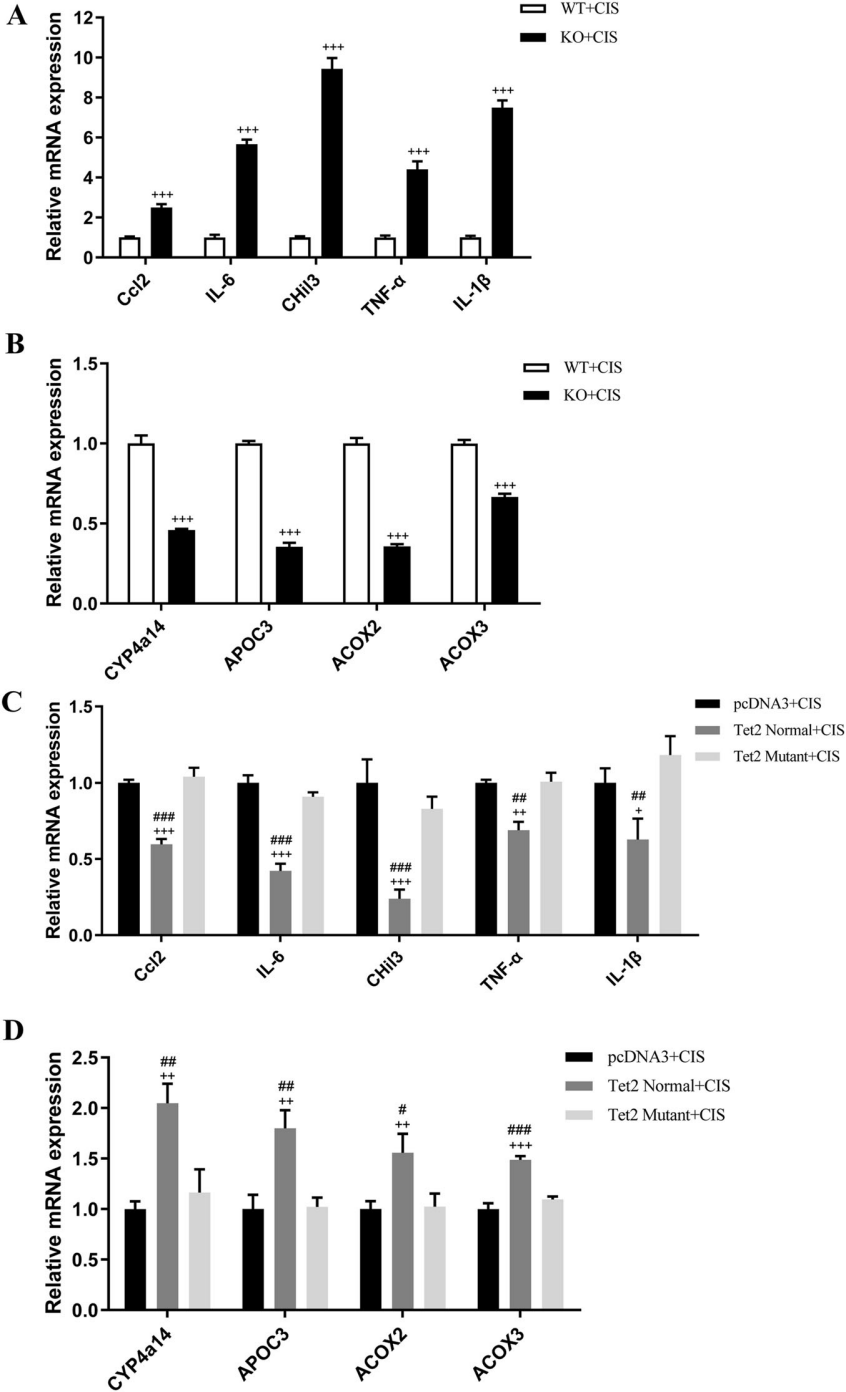
Quantitative analyses of the metabolic pathways and inflammatory genes in mice kidney (*n* = 3). **A**, **B** qPCR analysis of the indicated genes mRNA levels in WT and *Tet2*-KO mice kidney after cisplatin treatment. **C**, **D** qPCR analysis of the indicated genes mRNA levels in *Tet2* overexpression and control mice kidney after cisplatin treatment.

We conducted RNA-seq of the kidneys from cisplatin-injected KO and WT mice to explore the *Tet2* repression mechanism for cisplatin-induced renal injury. We observed 198 suppressed and 119 upregulated genes in the KO mice and compared them with the WT mice (Fig. 5A). A GO enrichment analysis in RNA-seq data showed highly enriched metabolic processes for organic acid, oxoacid, carboxylic acid, and the small molecule (Fig. 5B). The KEGG pathway analyses showed differential expressions of genes associated with metabolic pathways like arachidonic acid (AA) metabolism, xenobiotics cytochrome P450 metabolism, retinol metabolism, and PPAR signaling pathway (Fig. 5C). We then identified 13 downregulated genes (*P* ≤ 0.05; |fold change)| ≥1.5) related to the PPAR signaling pathway, *PPARGC1B*, *CYP4A14*, *APOC3*, and so forth (Fig. 5D). Among these genes, the transcription of *CYP4a14*, *APOC3*, *ACOX2*, *ACOX3*, and *Gyk* reduced at least two folds (Fig. 5E). Besides, a series of inflammation genes were upregulated in *Tet2*-KO mice (Fig. 5E). The qPCR analyses indicated an increased transcript of inflammatory cytokine genes (*Ccl2*, *IL-6*, *CHil3*, *TNF-α*, and *IL-1β*) and a decreased PPAR signaling pathway-related genes (*CYP4a14*, *APOC3*, *ACOX2*, and *ACOX3*) in the conditional KO mice compared to the WT mice after cisplatin injection (Fig. 6A, B). Besides, overexpression of *Tet2* in vivo could markedly suppress the transcription of inflammation genes (*Ccl2*, *IL-6*, *CHil3*, *TNF-α*, and *IL-1β*) and restore the reduced expression of *CYP4a14*, *APOC3*, *ACOX2*, and *ACOX3* in cisplatin-treated KO mice compared to overexpression of mutant *Tet2* or the control plasmid (Fig. 6C, D).

### Aggravation of cisplatin-induced AKI by deletion of *Tet2* gene is independent of tubular cell apoptosis

Cell apoptosis is a characteristic feature of cisplatin-induced AKI [18, 19], with the pro-apoptotic gene *Bax* playing a vital role [20, 21]. Therefore, we investigated if *Tet2* KO could affect the level of cisplatin-induced apoptosis in mouse kidney sections by conducting terminal deoxynucleotidyl transferase-mediated digoxigenin-deoxyuridine nick-end labeling (TUNEL) assay. Both KO and WT-mouse kidneys showed a similar apoptosis degree, although TUNEL-positive nuclei increased under cisplatin treatment (Fig. S1A, B). Immunohistochemistry analysis revealed no significant differences in BAX protein between cisplatin-administered WT and KO mice (Fig. S1C). Western blot results further indicated that *Tet2* KO did not affect the protein level of active caspase-3 (Fig. S2). Thus, we can infer that aggravation of cisplatin-induced AKI by *Tet2* gene deletion is independent of tubular apoptosis.

### Decreased *Tet2* expression is detected in both other mice AKI models and clinical patients

**Fig. 7:**
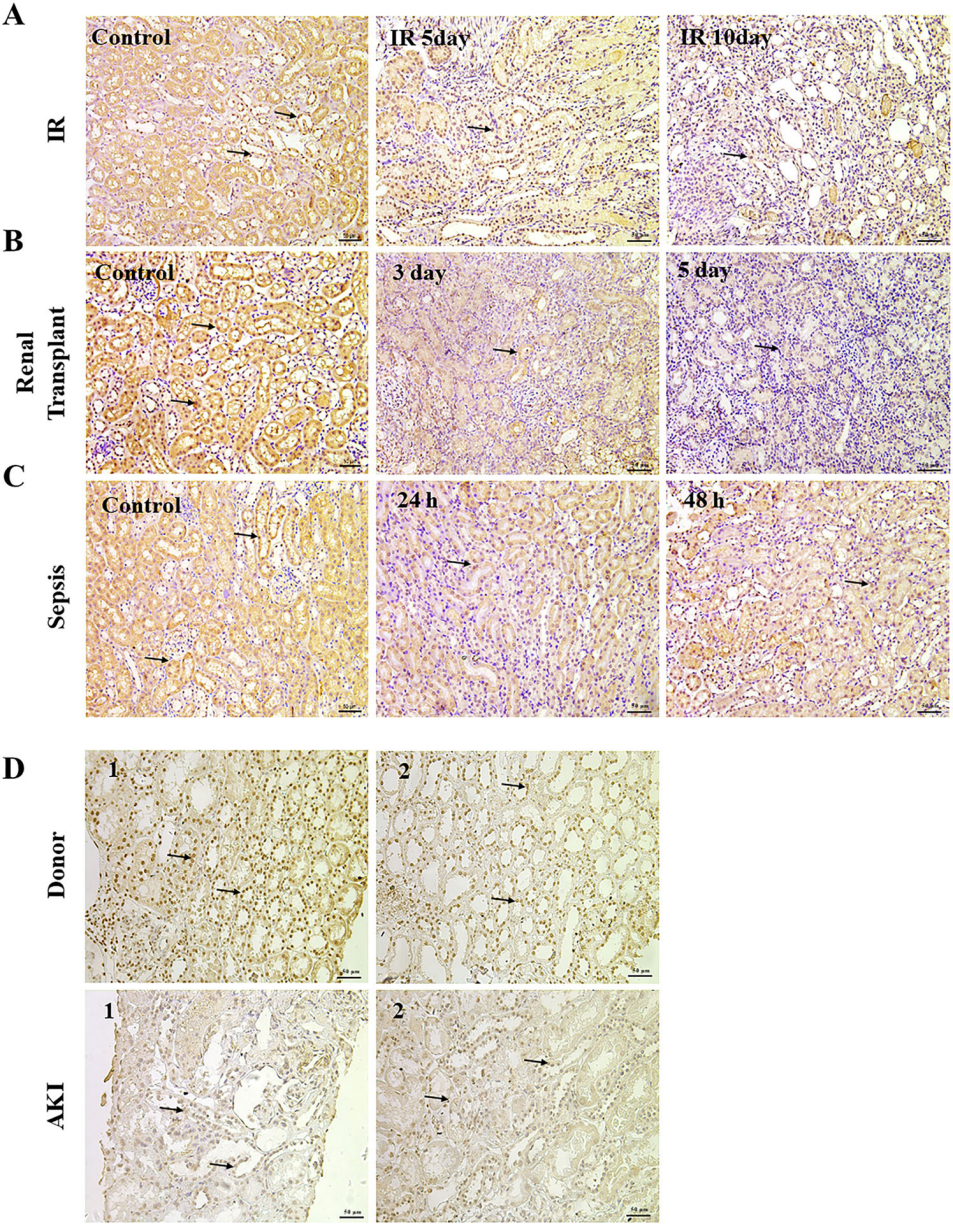
Tet2 expression is reduced in both experimental AKI models (*n* = 6) and clinical patients (*n* = 2). **A**–**C** Immunohistochemistry assays of different AKI models, Representative images of renal Tet2 protein staining are shown. Original magnification, ×200. **D** Immunohistochemistry of clinical renal biopsy tissues, original magnification, ×200. Small black arrows indicate a positive nucleus.

We observed reduced Tet2 expression in IR, renal transplant, and sepsis mice models at different time points, suggesting a possible important role of *Tet2* in AKI caused by other reasons (Fig. 7A–C). Immunohistochemistry of clinical renal biopsy tissues also confirmed lesser Tet2 proteins in AKI patients (Fig. 7D). Decreased Tet2 expression in various AKI models was a common observation; therefore, Tet2 might be a novel marker protein for predicting AKI in the future.

## Discussion

Tet methylcytosine dioxygenase (TET)’s primary function is its catalytic capability in oxidizing DNA 5mC to 5hmC through complete removal of methylated cytosine [22, 23]. DNA methylation changes have been implicated in diabetic nephropathy, kidney fibrosis, and chronic kidney disease [24]. However, very little research has been done on DNA hydroxymethylation and AKI relationship. Ning Huang and colleagues reported reduced levels of 5hmC and dramatically downregulated mRNA expression of *Tet2* in IR-injured kidneys [25]. The research indicated *Tet2* involvement in IR development, but the specific function of *Tet2* in this pathological process and its underlying mechanisms remained unclear. Therefore, in our study, we aimed to investigate the role of *Tet2* in cisplatin-induced AKI.

*Tet2*-KO increases cisplatin-induced tubular cell damage in vivo as evidenced by increased BUN, SCr, and pathological indexes. In addition, RNA-seq data suggested the protective role of *Tet2* by influencing multiple metabolic pathways such as arachidonic acid (AA) metabolism, cytochrome P450, other metabolic pathways, and the PPAR signaling pathway. Metabolic disorders impact the pathogenesis of renal dysfunction [26, 27]. Cytochrome P450 enzymes metabolize AA to four bioactive regioisomeric epoxyeicosatrienoic acids (EETs) and 20-hydroxyeicosatetraenoic acid (20-HETE) that mediate renal microcirculation, possess anti-inflammatory, and other renoprotective effects [28, 29]. The PPAR signaling pathway also plays a crucial protective role in acute renal tubular injury [30, 31]. PPARs are the primary regulators of fatty acid metabolism, and their derangement results in kidney injury [32–34]. Atf6α KO maintained expression of PPARα, and therefore reduced the apoptosis and lipid accumulation of proximal tubular cells following unilateral IR injury [33]. In addition, synthetic PPARα ligands, and PPAR-γ activation were proven to limit the expression of pro-inflammatory cytokines by attenuating NF-kB activity in cisplatin nephrotoxicity [35, 36]. Li et al. reported the anti-inflammatory effects of PPARα ligand in cisplatin-induced AKI by reducing renal endonuclease G [37]. Also, activation of PPAR-γ significantly contributed to protection against the IR-induced AKI because of its anti-inflammatory and anti-oxidant effects [38–40].

Our results showed that *Tet2* could regulate the PPAR signaling pathway, metabolism pathway, and inflammation response, thereby alleviating cisplatin-induced kidney injury. Renal tubular cell injury and necrosis, infiltration of inflammatory cells, especially macrophages, are the primary reasons for the occurrence and progression of AKI [41–43]. Therefore, in future experiments, *Tet2*-loxp mice should be mated with Kap-cre to dissect the functions of *Tet2* specifically in proximal tubule in cisplatin-induced AKI. Kap-cre expression is mainly detected in the proximal tubule cells [44]. Nakamura et al. explored the roles of transcription factor EB (TFEB) in AKI using proximal tubule-specific TFEB-KO mouse [44]. Suzuki et al. also demonstrated that Atg7 was essential for suppressing cell injury and apotosis using proximal tubule-specific Atg7-KO mouse [45]. The detailed roles of *Tet2* of renal tubular cells in cisplatin-induced AKI are worthy of further study. Overall, this study explored the function of *Tet2* in cisplatin-induced AKI for the first time; *Tet2* and DNA methylation may provide the potential therapeutic cues to treat cisplatin-induced AKI as well as other types of AKI.

## Materials and methods

### Animals

*Tet2*-floxed mice and EIIa-Cre mice were kindly provided by Dr. Ye Dan (Fudan University). The mice were housed in the Zhejiang University laboratory animal center. All experiments were approved by the Laboratory Animal Management and Ethics Committee of Zhejiang University per the Chinese Guidelines on the Care and Use of Laboratory Animals. *Tet2*-KO mice were generated by the Cre-loxP (Cre recombinase-locus of x-over, P1) system as described earlier (Fig. 2A). Mice genotypes were confirmed by tail-snip PCR amplification. Littermate control mice were used as controls in all animal-related experiments.

### Mouse model of cisplatin-induced AKI

An earlier research protocol was used to construct the cisplatin-induced AKI model [46]. Cisplatin (Selleck, S1166) was dissolved in 0.9% of saline at a concentration of 1 mg/mL. Male mice (8–10 weeks old, 20–25 g) were intraperitoneally injected with a single dose of cisplatin (22 mg/kg body wt) or an equal volume of saline. This dose was based on earlier studies [4, 47], and our preliminary experiments also proved that a lower dose did not produce a stable and significant acute tubular injury, while a higher dose was associated with more unpredictable mortality before 72 h after cisplatin injection in C57BL/6 mice. Mice ocular blood extraction was done at 0, 24, 48, and 72 h for BUN and creatinine measurements. The mice were sacrificed at 72 h after cisplatin administration, and the kidneys were either frozen with liquid nitrogen for qRT-PCR and Western blotting or fixed in 10% buffered formalin for histology/immunohistochemistry (IHC).

### Ischemia–reperfusion (IR) model

First, 50–60 mg/kg pentobarbital (5 mg/mL) was used to anesthetize the mouse by intraperitoneal (i.p.) injection [46]. The body temperature was maintained at 36.5–37 °C during the surgery. Second, the renal artery and vein were clamped by micro-aneurysm clips for a variable length of time to induce kidney injuries at varying severities. Successful ischemia was confirmed by a gradual darkening of the kidney (from red to dark purple). After ischemia, the clamp was removed at the desired time to achieve reperfusion; the kidney color immediately reverted to red. The mice were sacrificed at 5 and 10 days after IR, and the kidneys were fixed in 10% buffered formalin for further experimentation.

### Renal transplant model

Ectopic kidney transplantation was carried out as detailed previously [48, 49]. The kidneys from BALB/c mice were transplanted into C57BL/6 recipients. In brief, the renal artery and vein from BALB/c mice were anastomosed to the abdominal aorta and vena cava of the recipient mice, respectively. In addition, the donated ureter was attached to the recipient’s bladder. The transplanted kidneys were collected at 3 and 5 days after kidney transplantation.

### Mouse model of sepsis-associated AKI

The cecal ligation and puncture (CLP) model is the most frequently used model for its simplicity [46]. Intraperitoneal anesthesia of the mice was done as described in an earlier section. Ligation of the cecum from the distal to the ileocecal valve was made, followed by two needle-punctures to extrude the stool into the abdominal cavity. The CLP mice could produce typical bacterial peritonitis symptoms after 6 h. The mice were sacrificed at 24 and 72 h after CLP operations, and the kidneys were fixed in 10% buffered formalin for IHC detection.

### Overexpression of *Tet2* in vivo

Male C57BL/6 mice (8–10 weeks old) were given a continuous intravenous injection of WT and catalytic domain catalytic-dead *Tet2* overexpression plasmids (pScalps_Puro_mTet2 catalytic domain (#79554); pScalps_Puro_mTet2 catalytic domain HxD (#79611); Addgene) at 2 mg/kg body wt for 3 days (Fig. 5A) by using the hydrodynamic gene transfer technique [16, 50]. The control group was injected with an equal volume of saline. After plasmids administration, the mice were subjected to the mouse model of cisplatin-induced AKI as described earlier and were euthanized at 72 h after the model. The serum and the kidneys were collected for further experiments.

### Serum creatinine and BUN assay

Serum creatinine and BUN levels were detected by 7000i Automatic biochemical analyzer (FUJI). Serum creatinine was expressed as μmol/L and the BUN was expressed as mg/dL.

### Cell culture

HK-2 cells were obtained from ATCC, and the cells were cultured in DMEM medium supplemented with 10% heat-inactivated FBS, 1% penicillin, and streptomycin mixture. The cells plated in a 6-well plate overnight were treated with 10 µM cisplatin for 6–48 h to mimic acute injury. After the treatment, the cells were collected at different time points and subjected to various analyses.

### RNA-sequencing and bioinformatics analysis

RNA-sequencing (RNA-seq) was performed as described previously [41]. The total RNA was subjected to RNA-seq to determine mRNA expression patterns. Bioinformatics analyses including raw data process, differential gene expression analysis, gene ontology (GO), and Kyoto Encyclopedia of Genes and Genomes (KEGG) pathway analyses were conducted by Novogene Genomic Center.

### qPCR analysis

Total RNA was isolated from the kidneys or HK-2 cells using TRIzol Reagent (Invitrogen), and cDNA was synthesized using Reverse Transcription System Kit (Takara). The mRNA levels of various genes were quantified using CFX-96 Sequence Detection System (BIO-RAD). The detailed sequence of qPCR primers are listed in Table S1.

### Western blot analysis

Western blotting was performed as described in earlier research [51]. The primary antibodies used were as follows: anti-Tet2 (ab94580; Abcam), anti-GAPDH (10494-1-AP; Proteintech), and anti-caspase-3 antibody, active (cleaved) form (AB3623; Merk).

### Immunofluorescence staining

Immunofluorescence staining was performed as previously described [51]. The antibody used was anti-Tet2 (21207-1-AP; Proteintech).

### TUNEL staining assay

Apoptotic cell death in kidney tissue was determined by In Situ Cell Apoptosis Detection Kit (BBI Life Science), and the manufacturer’s instructions were followed for experimental operations.

### Histology and immunohistochemical staining

Immunohistochemical (IHC) staining was prepared in a traditional way described in earlier research [51], and human kidney biopsy samples were obtained from Kidney Disease Center at the First Affiliated Hospital, Zhejiang University. The kidney sections for IHC staining were prepared following the routine protocol. Antibodies used were anti-Tet2 (ab94580; Abcam) and anti-Bax (Sc-493; Santa Cruz Biotechnology).

### Statistical analyses

All data were expressed as mean ± SEM for statistical analyses. An unpaired *t*-test was used to analyze the group differences. A *P* value of ≤0.05 was considered statistically significant.

## Supplementary information

Supplmentary Figure and Table Legends

Supplemetnary Figure 1

Supplemetnary Figure 2

Table S1
