# Supplementary material for: DNA demethylase Tet2 suppresses cisplatin-induced acute kidney injury
Source: Cell Death Discov. 2021 Jun 17;7:167. doi: 10.1038/s41420-021-00528-7 (PMC8257623; doi:10.1038/s41420-021-00528-7)
Supplement: Supplementary file 1 — Supplmentary Figure and Table Legends [file 41420_2021_528_MOESM1_ESM.docx]

**Figure S1.** Tet2 knockout does not affect apoptosis in the mice kidneys following cisplatin treatment (*n*=3). (A) TUNEL assays of mice kidneys. Representative images of TUNEL staining are shown. Original magnification, X200. Positive staining is indicated by small white arrows. (B) Ten randomly selected fields (original magnification, X200) per kidney were required for counting TUNEL-positive cells. ###***P***＜0.001 versus WT mice; ******P***＜0.001 versus KO mice. (C) No significant difference of BAX expression was observed in both *Tet2* KO and WT mice after cisplatin treatment.

**Figure S2**. Western blot analysis of active Caspase-3 expression level. (A) Whole kidney lysate of different groups of mice were analyzed by western blot for Tet2 (*n*=2). GAPDH was used as a loading control. (B) Quantification of the bands’ intensity. Two independent experiments were performed. The data are the mean ± SEM; NS: Not Significant.

**Table S1**. qPCR primers used in this study*.* “Q” represents quantitative RT-PCR; “m” represents mouse; “h” represents homo sapiens; “F” represents forward primers; “R” represents reverse primers.
