# Supplementary figures and images for: DNA demethylase Tet2 suppresses cisplatin-induced acute kidney injury
Source: Cell Death Discov. 2021 Jun 17;7:167. doi: 10.1038/s41420-021-00528-7 (PMC8257623; doi:10.1038/s41420-021-00528-7)

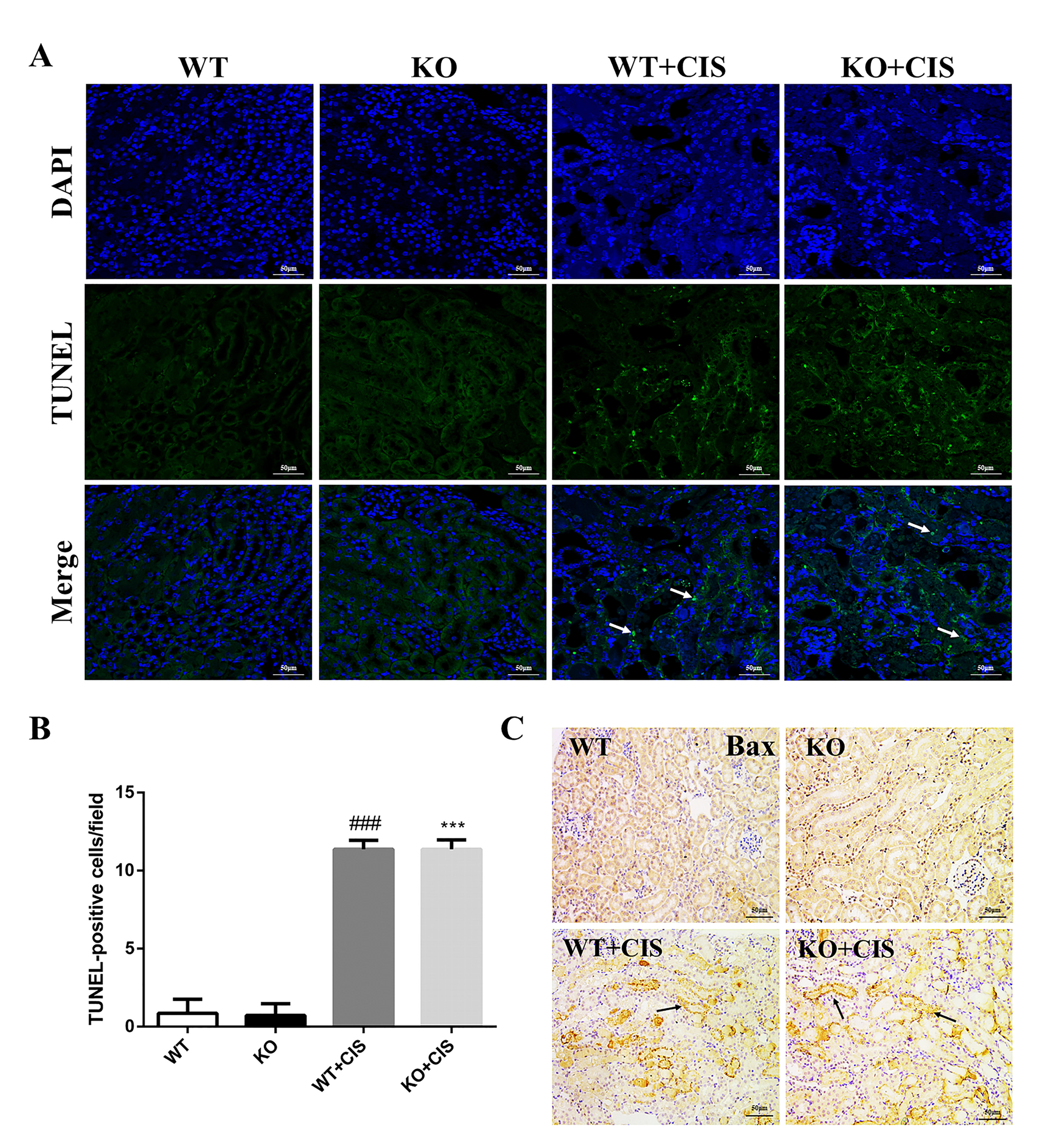

Supplement: Supplementary file 2 — Supplemetnary Figure 1 [file 41420_2021_528_MOESM2_ESM.tif]

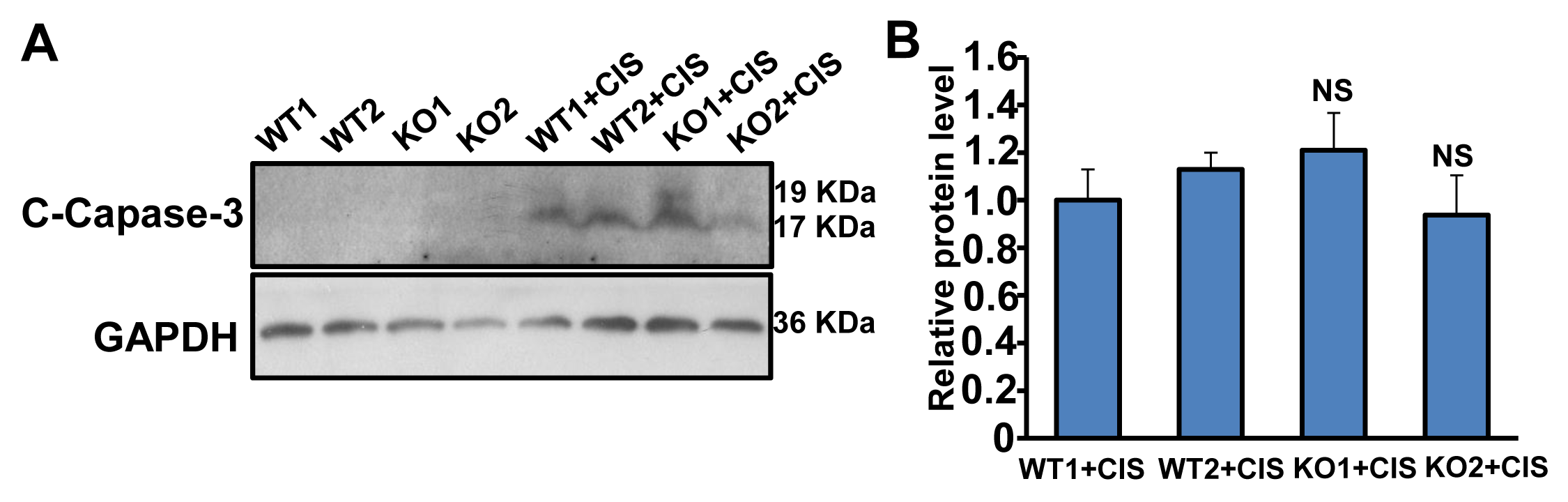

Supplement: Supplementary file 3 — Supplemetnary Figure 2 [file 41420_2021_528_MOESM3_ESM.tif]
