# Supplementary material for: DNA demethylase Tet2 suppresses cisplatin-induced acute kidney injury
Source: Cell Death Discov. 2021 Jun 17;7:167. doi: 10.1038/s41420-021-00528-7 (PMC8257623; doi:10.1038/s41420-021-00528-7)
Supplement: Supplementary file 4 — Table S1 [file 41420_2021_528_MOESM4_ESM.doc]

Table S1. qPCR primers used in this study*.* “Q” represents quantitative RT-PCR; “m” represents mouse; “h” represents homo sapiens; “F” represents forward primers; “R” represents reverse primers.

| **Primer** | **Sequence** |
| --- | --- |
| GAPDH_mQ_F | GGTGAAGGTCGGTGTGAACG |
| GAPDH_mQ_R | CTCGCTCCTGGAAGATGGTG |
| GAPDH_hQ_F | TCAAGGCTGAGAACGGGAAG |
| GAPDH_hQ_R | TGGACTCCACGACGTACTCA |
| TET1_mQ_F | ATCTGGGGCCATCCAAGTC |
| TET1_mQ_R | TGTGTGAACCTGATTTATTGTGGT |
| TET2_mQ_F | GAAAAGGCCCGGAAAGAGGA |
| TET2_mQ_R | GCCCTGTGACCTGAGTGAAA |
| TET3_mQ_F | CTGTGGTCGGACAGTGAACA |
| TET3_mQ_R | TGGGCTGGTTGAGGTTCTTG |
| TET2_hQ_F | ATGGCTTGGCTCTTTGGGAA |
| TET2_hQ_R | GCAGGTAAGTGGGCTCTGAA |
| KIM1_mQ_F | GCTGCTACTGCTCCTTGTGA |
| KIM1_mQ_R | GGAAGGCAACCACGCTTAGA |
| NGAL_mQ_F | GGCCAGTTCACTCTGGGAAA |
| NGAL_mQ_R | TGGCGAACTGGTTGTAGTCC |
| CCL2_mQ_F | CATCCACGTGTTGGCTCA |
| CCL2_mQ_R | GATCATCTTGCTGGTGAATGAGT |
| IL6_mQ_F | ACCCCAATTTCCAATGCTCTC |
| IL6_mQ_R | ATGGTCTTGGTCCTTAGCCAC |
| CHIL3_mQ_F | ATCTATGCCTTTGCTGGAATGC |
| CHIL3_mQ_R | TGAATGAATATCTGACGGTTCTGAG |
| TNFA_mQ_F | CTGTAGCCCACGTCGTAGC |
| TNFA _mQ_R | TTGACATCCATGCCGTTG |
| IL1B_mQ_F | CTGCAGCTGGAGAGTGTGGAT |
| IL1B _mQ_R | GCTTGTGCTCTGCTTGTGAG |
| CYP4A14_mQ_F | TTTAGCCCTACAAGGTACTTGGA |
| CYP4A14_mQ_R | GCAGCCACTGCCTTCGTAA |
| APOC3_mQ_F | TACAGGGCTACATGGAACAAGC |
| APOC3_mQ_R | CAGGGATCTGAAGTGATTGTCC |
| ACOX2_mQ_F | AACCCAGGGGATCGAGTGT |
| ACOX2_mQ_R | CGCAGCTCAGTGTTTGGGAT |
| ACOX3_mQ_F | CAGAATGGTGTGCTAGAGCGT |
| ACOX3_mQ_R | AGCCTGTCGGCTACAGATTTG |
